# In-Hospital Onset of Pseudothrombocytopenia Days before Surgery

**DOI:** 10.1155/2018/4726036

**Published:** 2018-12-31

**Authors:** Luiz Souza, Luiz Amorim, Ana Pereira

**Affiliations:** ^1^Faculdade Técnico-Educacional Souza Marques (FTESM), Hospital Miguel Couto, Rio de Janeiro, Brazil; ^2^Instituto Estadual de Hematologia Arthur de Siqueira Cavalcanti (HEMORIO), Rio de Janeiro, Brazil; ^3^Instituto Nacional de Câncer (INCA), Rio de Janeiro, Brazil

## Abstract

Automated cell counters often produce spuriously low platelet counts due to laboratory artifacts. These* in vitro* phenomena may lead to erroneous treatments, surgical delays, and unnecessary platelet transfusions. An overlooked case of newly developed anticoagulant-induced platelet aggregation diagnosed in a preoperative visit is discussed and diagnostic clues are presented.

## 1. Introduction 

Anesthesiologists base numerous important decisions on electronic counted cells. Hematology analyzers save priceless time and reduce human errors provided that occasional automated warning alarms called “*flags*” are verified. Devoid of clinical significance if promptly recognized, pseudothrombocytopenia (PTCP) or platelet aggregation induced by ethylenediaminetetraacetic acid (EDTA) at room temperature is an important mishap especially in patients seen by anesthesiologist's just before nonelective surgery. Ordering simple laboratory procedures is sufficient to unravel most cases of anticoagulant-induced* ex vivo* platelet aggregation as reported herein. PTCP and related conditions should always be included in the differential diagnoses of unexpected thrombocytopenia.

## 2. Case Report 

**Figure 1:**
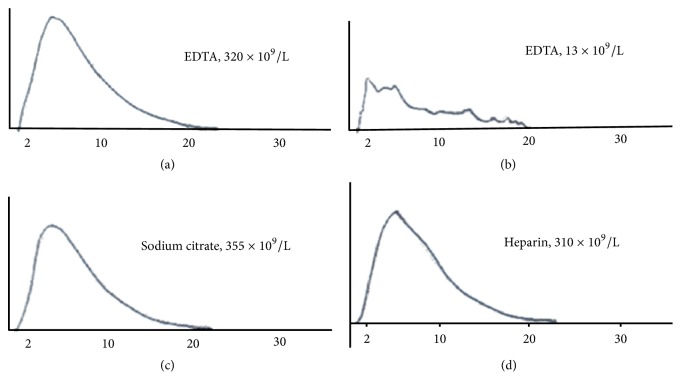
Platelet counts and histograms within normal limits in EDTA on hospital admission (a), in sodium citrate (c), and in heparin (d). The EDTA aliquot obtained six days after admission presented multiple peaks and a depressed curve's height suggesting respectively platelet aggregation and thrombocytopenia (b). X-axis in femtoliters.

**Figure 2:**
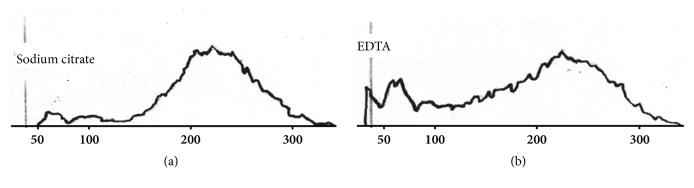
WBC histograms of a sample drawn six days after admission. A left peak in the ghost zone (<50 femtoliters) suggesting platelet clumping is absent in sodium citrate (a) but present in EDTA (b). X-axis in femtoliters.

A 61-year-old man with severe soft tissue infection in one leg was admitted to an emergency hospital. Ceftriaxone, clindamycin, and prophylactic enoxaparin were added to his previous medications (enalapril and simvastatin). Four days later the leg was deemed beyond salvage. Preoperative laboratory findings were unremarkable except for thrombocytopenia (*Coulter*® LH 750 Analyzer, Beckman Coulter Life Sciences). Platelet counts had dropped from 320x10^9^/L to 8x10^9^/L in EDTA. No* platelet increment* was observed* after three *full-dose platelet transfusions over the next* two days. Accompanying flags and blood smears were not mentioned in the medical chart. A* consultant anesthesiologist suspected PTCP and a blood sample in EDTA, sodium citrate, and heparin was processed at room temperature. Further workup was unnecessary. Platelet counts were 13x10^9^/L in EDTA but 355x10^9^/L in sodium citrate and 310x10^9^/L in heparin.* Thrombocytopenia* and* platelet aggregates* were flagged only in the EDTA aliquot. Abnormal platelet histogram and white blood cell (WBC) also suggested* in vitro* platelet clumping (Figures 1 and 2). Amputation under spinal anesthesia was carried out uneventfully.

## 3. Discussion

PTCP was first described in 1969 [1]. The phenomenon is widely reported but seldom discussed in medical textbooks. Clumping usually increases as a sample's temperature decreases [2]. Prevalence varies from 0.04% to 2.1% in patients treated with abciximab but may be as high as 16% in selected outpatients [3–5]. No predisposing condition exists. Onset and resolution when it occurs are unpredictable. Platelet function and surgical procedures under hypothermia are normal. Transient transplacental neonatal PTCP is possible but platelet donation is not contraindicated.

Anticoagulants sometimes alter cytoadhesive glycoprotein IIb*/*IIIa receptor complexes* in vitro *and expose cryptic epitopes on platelets' surface. In more than 80% of PTCP, natural autoantibodies against these sites are present and contribute to aggregation [6]. Optimal aggregation temperature depends on the anticoagulant used. A specimen may present clumping at body temperature in sodium citrate and at room temperature or lower (*cold agglutinins*) in EDTA [6]. Antigen-binding fragments (*Fab*) of some monoclonal antibodies are also implicated [4, 7]. Sodium citrate and/or heparin coexist with PTCP in 6% to 16% of patients but five substances were implicated in a single case [3, 6, 8]. Response to anticoagulants may change spontaneously over time [9].

Failure to perform confirmatory blood smears and/or to interpret subjective findings properly explains unnoticed cases of PTCP. Partially clotted or overheated specimens are usually discarded in the preanalytical phase.* Thrombocytopenia* and* platelet aggregates *(*MP* in some instruments) are usually flagged. Retesting a sample in alternative anticoagulant substances will identify most cases in a few minutes. Special anticoagulants (e.g., magnesium-based salts) are seldom necessary unless accurate counts are desired. Clumps vary in size generating misleading flags (e.g.,* giant platelet*) and multiple-peak histograms (Figure 1). Similar histograms are seen in partially coagulated samples and in platelet anisocytosis (e.g., after chemotherapy or platelet transfusions). Left peaking WBC histograms may also indicate PTCP, especially when a sample aliquoted in different anticoagulant substances (Figure 2). Anticoagulant-independent spurious thrombocytopenia is quite rare [3, 10]. It is caused by true giant platelets or leukocyte-platelet aggregates (e.g., satellitism).

## 4. Conclusions

Pseudothrombocytopenia is still overlooked six decades after its description. Anesthesiologists and transfusion services must work in concert with medical laboratories to ensure that unexpected instrument-derived platelet counts are confirmed before embarking on futile platelet transfusion strategies.
